# Gamma-Aminobutyric Acid (GABA) as a Dietary Strategy for Enhancing Temperature Stress Resilience in Aquaculture Species

**DOI:** 10.3390/ijms262010233

**Published:** 2025-10-21

**Authors:** Abayomi Oladimeji Ogun, Mohammad Moniruzzaman, Hyuncheol Jeon, Haham Kim, Deni Aulia, Junhyeok Hur, Sooa Yoon, Suhyun Lee, Taesun Min, Seunghyung Lee

**Affiliations:** 1 Major of Aquaculture and Applied Life Sciences, Division of Fisheries Life Sciences, Pukyong National University, Busan 48513, Republic of Korea; abayomi.ogun@yahoo.com (A.O.O.); conjp@naver.com (H.J.); haham7@naver.com (H.K.); damursalin@gmail.com (D.A.); junhyeok1999@naver.com (J.H.); dbshelena@naver.com (S.Y.); su8842@naver.com (S.L.); 2 Department of Animal Biotechnology, Jeju International Animal Research Center, Sustainable Agriculture Research Institute (SARI), Jeju National University, Jeju 63243, Republic of Korea; monir1983@jejunu.ac.kr (M.M.); tsmin@jejunu.ac.kr (T.M.); 3 Feeds and Foods Nutrition Research Center, Pukyong National University, Busan 48547, Republic of Korea

**Keywords:** sustainability, climate change, stress relief, global warming

## Abstract

The sustainability of aquaculture is increasingly threatened by rising ocean temperatures occasioned by the continued prevalence of global warming, which can have severe consequences for fish health and productivity. Fish, as ectothermic organisms, are susceptible to temperature fluctuations and prolonged exposure to extreme temperatures can lead to physiological disruptions, including altered metabolic rates, oxidative stress, and immune suppression, ultimately affecting their growth and reproductive success. In response, several strategies, including dietary supplementation, have been proposed to alleviate temperature stress in aquaculture. One such supplement, gamma (γ)-aminobutyric acid (GABA), a non-proteinogenic amino acid, has garnered attention for its potential to enhance stress resilience in aquatic species. In this review, we examine the physiological responses of fish to temperature stress and evaluate the role of GABA in alleviating non-temperature stress. By synthesizing the available evidence, we aim to highlight the potential of GABA as a dietary supplement to improve the resilience of farmed fish to temperature fluctuations, ultimately contributing to sustainable aquaculture in the face of climate change. GABA acts as an inhibitory neurotransmitter in the central nervous system, promoting relaxation and reducing stress. We not only spotlight GABA’s role in the central nervous system, where it has been shown to modulate stress responses by enhancing antioxidant defenses, improving growth performance, and boosting disease resistance, but also emphasize the limited exploration of its potential to mitigate temperature stress in some aquaculture species, particularly economically important fish like olive flounder. Finally, in this review, we provide additional insights into how GABA might help mitigate temperature stress by identifying factors that may influence its supplementation, thereby laying the groundwork for future research on its use as a potential tool for mitigating temperature stress in aquaculture species.

## 1. Introduction

Aquaculture sustainability is increasingly threatened by rising ocean temperatures [1]. Ectothermic organisms, like fish, are highly sensitive to fluctuations in water temperature [2]. While moderate increases in temperature can enhance metabolic rates and promote growth, prolonged exposure to extreme temperatures can have severe consequences, depending on the intensity and duration of exposure. Such conditions can lead to a cascade of physiological disruptions, including altered metabolic rates, protein denaturation, oxidative stress, and impaired immune function [2,3]. These disruptions not only threaten fish survival but also significantly reduce growth rates, reproductive success, and overall aquaculture productivity [4,5,6]. As climate change is projected to continue, countries such as Republic of Korea, where climate change is accelerating at a rate faster than the global average, are expected to experience temperature increases of 1–4 °C above the global average by 2100 [7,8,9]. Consequently, mitigating temperature stress is crucial for sustaining the productivity and profitability of aquaculture systems [3,10].

Various strategies have been proposed to alleviate temperature stress in aquaculture [11]. Among these, dietary supplements that bolster fish resilience to stress have shown significant potential to improve resilience under stress conditions [12]. One such supplement that has garnered considerable attention is gamma (γ)-aminobutyric acid (GABA), a non-proteinogenic amino acid [13]. GABA plays a vital role in managing anxiety and stress in vertebrates by functioning as an inhibitory neurotransmitter in the central nervous system (CNS), promoting relaxation and reducing stress [14,15]. Beyond its role in the CNS, GABA is increasingly recognized for its broader physiological effects, particularly its ability to modulate stress responses in many aquatic species under various non-temperature stress conditions. For example, whiteleg shrimp (*Litopenaeus vannamei*) fed a GABA-supplemented diet exhibited enhanced antioxidant status, improved growth performance, better feed efficiency, and increased disease resistance against *Vibrio alginolyticus* during bacterial challenge stress [12,15]. Similarly, white leg shrimp receiving a low fishmeal diet supplemented with GABA not only showed improved growth but also elevated levels of endocrine hormones, such as insulin and neuropeptide Y, along with enhanced tolerance to both nutritional and ammonia stress [14,15]. In another study, GABA supplementation in olive flounder resulted in increased growth and enhanced disease resistance against *Streptococcus iniae* [13,15]. Collectively, these findings underscore GABA’s potential as a dietary supplement to improve stress resilience and optimize performance in aquaculture species.

Despite the promising effects of GABA under various non-temperature stress conditions, its efficacy in mitigating temperature-induced stress remains insufficiently explored, particularly in economically significant species such as olive flounder, a key aquaculture species in Korea. Ogun et al. [16] reported no significant improvements in thermal stress tolerance following GABA supplementation. However, they emphasized that GABA’s effectiveness may depend on several interacting factors. This perspective aligns with the understanding that temperature stress, like other stressors, activates similar physiological pathways, including the hypothalamus–pituitary–interrenal (HPI) axis, which triggers the release of glucocorticoids (primarily cortisol) and catecholamines. It also leads to the overproduction of reactive oxygen species (ROS), oxidative damage, and immune suppression [17,18]. These shared stress mechanisms suggest that GABA could alleviate temperature stress by addressing these physiological disruptions. For example, GABA has been shown to reduce cortisol levels and regulate the stress response, which may help prevent the prolonged activation of the HPI axis, a critical driver of temperature stress. Additionally, GABA’s antioxidative properties could counteract the oxidative damage caused by elevated metabolic rates, reducing ROS production and promoting cellular integrity [19]. Furthermore, GABA’s immune-boosting effects may help mitigate the immunosuppression often associated with temperature stress, enhancing fish resistance to pathogens [20,21,22].

Given these shared stress pathways, GABA may still offer therapeutic potential in alleviating temperature stress. GABA has been shown to modulate the HPI axis by reducing cortisol secretion and attenuating the stress response, potentially preventing prolonged endocrine activation. Its antioxidant properties can also help counteract ROS accumulation and oxidative tissue damage, thereby preserving cellular integrity. Moreover, GABA’s immunomodulatory effects may mitigate the immunosuppressive consequences of thermal stress, enhancing disease resistance in fish. Ogun et al. [16] therefore suggest that, although their findings were inconclusive, further investigation into the contextual factors influencing GABA’s efficacy could reveal its potential as a dietary intervention for temperature stress mitigation in aquaculture.

This potential of GABA to mitigate temperature stress in aquaculture is particularly timely, given the growing concerns about climate change and its impact on aquatic ecosystems. By harnessing GABA’s stress-modulating properties, aquaculture operations may improve the resilience of farmed fish to temperature fluctuations, thereby reducing adverse effects on growth, survival, and overall productivity.

This review, therefore, aims to assess the potential of GABA as a dietary supplement for mitigating temperature-induced stress in aquaculture species. Specifically, we examine the physiological mechanisms underlying temperature stress responses in fish and review evidence of GABA’s role in alleviating non-temperature stress across diverse aquatic species. By synthesizing current findings, we aim to better understand how GABA might be leveraged to combat temperature-related stress. Additionally, this review identifies key factors that may influence the effectiveness of GABA supplementation and outlines research gaps and future directions for optimizing its use in climate-resilient aquaculture systems.

## 2. Physiological Adaptations of Fish to Temperature Stress

**Figure 1:**
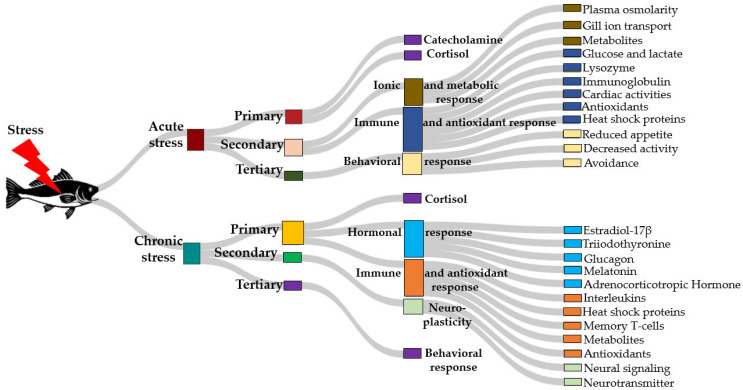
Sankey diagram illustrating the progression of acute and chronic stress responses in fish. Stress triggers primary, secondary, and tertiary responses, which cascade into physiological, immune, metabolic, and behavioral changes. Key outcomes include oxidative stress regulation, hormonal modulation, and behavioral adaptation, highlighting the interconnected nature of stress mechanisms in aquatic organisms.

When fish are subjected to temperature stress, their physiological responses are classified as either acute or chronic, depending on the duration and severity of the temperature change [23] (Figure 1). Acute temperature stress refers to sudden, short-term fluctuations in temperature [2]. However, when these disturbances are prolonged, they lead to chronic stress, causing a sustained loss of homeostasis, to which adaptation becomes impossible. In response to both types of stress, fish first activate brain centers, triggering the release of significant amounts of catecholamines and corticosteroids. Secondary reactions, such as the release of hormones into the bloodstream and tissues, metabolic disruption, energy mobilization, and the regulation of hydromineral balance, follow this primary response. Tertiary responses, which affect the organism and population levels, include growth inhibition and reduced reproductive and immune performance are triggered upon prolonged exposure [24,25,26,27,28].

Acute temperature fluctuations can lead to sublethal physiological responses and, in extreme cases, mortality based on the degree or intensity [28,29]. Elevated cortisol levels in exposure to acute temperature increases have been reported in various fish species, alongside secondary stress responses such as increased cardiac activity, elevated glucose and lactate levels, changes in blood osmolality, and other hematological and physiological alterations [2,28] (Table 1). Chronic or repeated stress leads to the reallocation of energy substrates, which could become detrimental to the normal function of the fish [2].

### 2.1. Nervous System-Mediated Endocrine Adaptation

Temperature plays a crucial role in various aspects of the nervous system, including brain organization, neurogenesis, and overall function [28,30,31]. Temperature changes can significantly disrupt the functioning of the nervous system and its components. Species-specific variations in temperature plasticity may limit fish’s ability to adapt to the anticipated effects of global warming. Additionally, these neural changes are likely to be compounded by other environmental stressors, potentially affecting essential functions such as feeding, immunity, behavior, and reproduction, which are key factors in aquaculture production. The release of catecholamines triggers physiological responses that enhance oxygen transport, respiration, energy mobilization, and cardiac activity [24,28,32,33].

Exposure to elevated temperatures has been shown to increase cortisol levels in various species such as Atlantic cod (*Gadus morhua*) [28,34]. European seabass (*Dicentrarchus labrax*) [28,35]. Nile tilapia (*Oreochromis niloticus*) [28,36], and olive flounder (*Paralichthys olivaceus*) [28,37]. Both acute and chronic temperature fluctuations disrupt cardiac rhythms due to alterations in ionic balance [38]. For example, elevated temperatures impair cardiac function in species such as Atlantic salmon (*Salmo salar*) [39,40], sparid (*Chrysoblephus laticeps*) [41], and rainbow trout (*Oncorhynchus mykiss*) [42], as well as reducing blood flow to critical organs such as the brain, liver, kidney, and intestine in rainbow trout [28].

**Table 1:** Effects of temperature on fish physiology.

Species	Temperature	Duration	Tissues and Organs Assessed	Effects	Conclusion	References
*Alosa sapidissima*	24 °C (Low), 27 °C (Mid), and 30 °C (High)	NS	Gills, liver	SOD 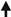 CAT 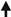 CORT 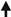 MDA 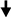 ALP 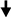 LDH 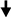	Temperature stress altered the structural characteristics of the gills, induced increased activities of the antioxidant enzymes SOD and CAT and the secretion of cortisol to increase heat tolerance.	[43]
*Dicentrarchus* *labrax*	8 °C and 32 °C	30 days	Plasma, Blood, liver	TG 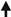 Lactate 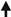 Liver size 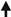 GLU 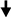 Erythrocyte **~**	Temperature fluctuation caused significant and continuing changes in hemato-biochemical indicators and erythrocyte peripheral and nuclear structure	[44]
*Cyprinus carpio*	32 °C	48 h	Liver, intestine, gill, Blood	SOD 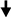 CAT 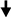 GPx 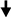 NBT 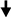 LZM 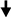 MDA 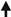	Exposure to thermal stress increased oxidative stress and diminished the immunity of common carp reared in hyper salinity conditions	[45]
*Notothenia coriiceps* and *Notothenia rossii*	4 °C	1–6 days	Gill, Liver, and Brain	GPx 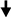 SOD 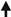 MTLP 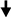 LPO 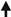 GSH 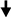 GCL 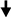 Erythrocytes 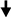	Sensitivity to ocean warming by tissue oxidative damage associated with thermal stress	[46]
*Oncorhynchus mykiss*	25 °C	NS	spleen, serum	MRB 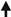 SOD 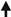 MDA 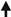 *hsp47* 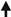	Heat shock causes cell injury, induces oxidative damage and promotes SERPINH1 mRNA	[47]
*Oncorhynchus mykiss*	25 °C	12 h	gill, liver, spleen, heart, brain, and head kidney	CORT 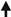 *hsp40* 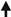 *hsp90β* 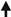	Cells may be damaged under heat stress with increasing AKP activity and elevating CORT	[20]
*Sander lucioperca*	28 °C, 32 °C, 34 °C and 36 °C	2 h	blood, liver, brain, gills, heart, spleen, stomach, intestine, and muscle tissue	SOD 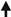 CAT 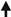 GPx 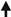 TBARS 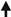 H_2_O_2_ 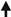 ALT 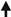 AST 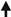 LDL 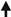 HDL 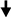 TG 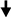 CHO 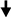	Heat stress significantly affected the physiological and biochemical activities of pikeperch. Our results showed that heat stress elevated the levels of ROS, then caused antioxidant defenses and an immune response	[48]
*Oncorhynchus mykiss*	25 °C	for 2, 4, 8, and 12 h	gill	*hsp30* 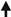	*hsp30* mRNA expression in rainbow trout was induced by heat stress and responded in a time- and tissue-specific manner.	[49]

**SOD:** Superoxide dismutase; **CAT:** Catalase; **TG:** Triglycerides; **GLU:** Glucose; **GCL:** Glutamate cysteine ligase; **GSH:** Glutathione; **LPO:** Lipid peroxidation; **MTLP:** Metallothionein-like proteins; **LZM:** Lysozyme; **NBT:** Nitroblue tetrazolium; **GPx:** Glutathione peroxidase; **LDH:** Lactate dehydrogenase; **ALP:** Alkaline phosphatase; **MDA:** Malondialdehyde; **CORT:** Cortisol; Represents an increase in the corresponding parameters; **~:** Represents abnormalities; **NS**: Not stated (unknown). **MRB**: Macrophage respiratory burst; MDA: Malondialdehyde; **SOD**: Superoxide dismutase; **GPx:** Glutathione peroxidase; **CAT:** Catalase; **TBARS:** Thiobarbituric acid; **H_2_O_2_**: Hydrogen peroxide; **ALT:** Alanine transaminase; **AST**: Aspartate transaminase; **LDL**: Low density lipoprotein; **HDL**: High density lipoprotein; **TG**: Triglycerides; **CHO:** Cholesterol; ***hsp*:** Heat shock protein 30, 40, 90, *β*(beta); **SERPINH1 mRNA:** Serpin family H member 1 protein; **AKP**: Alkaline phosphatase: **ROS:** Reactive oxygen species; 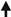 Represent an increase in the corresponding parameters; 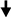 Represents a decrease in the corresponding parameters.

Chronic exposure to temperature stress triggers neuroendocrine responses in fish, activating the release of corticosteroids and catecholamines, which can lead to secondary stress reactions [24]. For instance, *Olive flounder* exposed to 28 °C and 30 °C for two weeks exhibited significantly higher plasma cortisol and acetylcholinesterase (AChE) levels compared to fish maintained at 20 °C, signaling a stressful environment [28,50]. Similarly, rainbow trout subjected to an acute temperature shock (from 13 °C to 25 °C for 4 h) experienced a marked increase in catecholamine release, reduced arterial oxygen, and heightened CO_2_ levels [28]. High-temperature exposure (18 °C) in Sturgeon (*Acipenser fulvescens*) led to an increase in whole-body cortisol content when compared to fish kept at 10 °C [28]. Key neurotransmitters such as dopamine (DA), noradrenaline (NE), and particularly serotonin (5-HT) also play significant roles in maintaining homeostasis. In juvenile Chinook salmon (*Oncorhynchus tshawytscha*), exposure to 16.4 °C and 19 °C for 14 days (from an acclimation temperature of 11 °C) resulted in increased levels of estradiol-17β (E2), testosterone, triiodothyronine, and thyroxine in comparison to the control group at 11 °C [28,51]. However, a decrease in dopamine, gonadotropin-releasing hormone receptor 2A, and growth hormone 1 (GH1) was observed in fish acclimatized to temperatures of 16.4 °C and 19 °C [51]. Similarly, Rainbow trout exhibited increased levels of brain estradiol-17β, cortisol, triiodothyronine, and thyroxine when acclimatized to 16 °C and 19 °C over an 11-day period [28]. Meanwhile, in Nile tilapia, temperature changes significantly reduced serum estradiol and cortisol levels [15,36].

### 2.2. Antioxidant Adaptation Mechanisms

Oxygen is vital for metabolic activities in fish; however, excessive oxygen can lead to cellular damage and even cell death through the generation of reactive oxygen species (ROS). These ROS can cause oxidative stress, which disrupts cellular homeostasis, leading to various forms of damage, including protein carbonylation, DNA damage, lipid peroxidation, and oxidation [52]. To counteract ROS-induced damage, fish possess both enzymatic and non-enzymatic antioxidant systems, which play crucial roles in mitigating oxidative stress [52,53,54]. These antioxidants work together to neutralize ROS, maintaining cellular integrity and function. However, during periods of temperature stress, the balance between ROS production and antioxidant defense can be disrupted, leading to oxidative damage when the antioxidant systems are insufficient [55]. This can be particularly detrimental in aquaculture systems where fish are exposed to varying and often extreme temperature fluctuations. The antioxidant response of fish during temperature stress involves complex interactions between enzymatic and non-enzymatic mechanisms [54,55,56]. The activities of enzymatic antioxidants, including superoxide dismutase (SOD), catalase (CAT), and glutathione peroxidase (GPx), constitute the first line of defense, playing key roles in neutralizing ROS. Meanwhile, non-enzymatic antioxidants, such as vitamins C and E, glutathione (GSH), and metabolites (including bilirubin and melatonin) are the second line of defense [52], contributing to the detoxification of ROS and reducing oxidative damage at the cellular level [52,53,55]. When the antioxidant defense systems in fish become overwhelmed due to prolonged temperature stress, cellular damage can accumulate, impairing overall fish health and productivity. The localization and activity of antioxidant enzymes and non-enzymatic antioxidants in response to temperature stress are critical factors in determining the resilience of fish to temperature-induced oxidative damage [52].

Acute temperature stress triggers a substantial antioxidant response in fish, as they attempt to mitigate oxidative damage caused by elevated temperatures [57]. For instance, in gilthead seabream (*Sparus aurata*) exposed to temperatures above 24 °C for 48 h, an increase in the activities of superoxide dismutase (SOD), glutathione-S-transferase (GST), catalase (CAT), lipid peroxidation (LPO), and cytochrome P450 (CYP1A) was observed [28,29]. Similarly, in fathead minnow *Pimephales promelas*, a significant increase in the levels of glutathione (GSH), glutathione reductase (GR), CAT, and GST was observed after 3 h of temperature stress at 32 °C [58]. Additionally, chronic exposure to temperature stress also activates the antioxidant defense mechanisms in fish. European seabass exposed to high temperatures (28 °C, 32 °C, and 33.3 °C) for 14 days demonstrated higher activities of GST, CAT, LPO, and malondialdehyde (MDA) compared to fish maintained at 24 °C [59,60,61]. For these species, significantly elevated antioxidant responses were observed at temperatures >32 °C compared to 16 °C [28,60]. Furthermore, Medaka (*Oryzias melastigma*) exposed to 25 °C for 3 weeks exhibited increased activities of glutathione peroxidase (GPx), CAT, SOD, GR, and LPO compared to fish kept at 18 °C [28,62].

### 2.3. Molecular and Metabolic Adaptations

Many studies have investigated how temperature stress influences the metabolic and molecular stress responses in fish. For instance, higher temperatures impair metabolic functions, as observed in striped catfish (*Pangasianodon hypophthalmus*) exposed to 36 °C for 28 days, resulting in increased blood glucose levels compared to fish at 24 °C, 28 °C, and 32 °C [28,63]. In addition to metabolic disruptions, temperature stress triggers cellular regulatory mechanisms aimed at mitigating damage. Heat shock proteins (hsps), particularly *hsp70*, function as molecular chaperones, preventing protein denaturation and aiding in cellular repair during elevated temperature stress [25,28,64,65,66]. Hsps are integral to long-term stress adaptation, as their synthesis increases following initial exposure to stress, helping to protect cells and tissues from structural damage during subsequent exposures [59,67,68]. A significant upregulation of *hsp70* has been reported in olive flounder exposed to 28 °C and 30 °C for 2 weeks [50]. In European seabass, *hsp70* expression was markedly increased after 30 days of acclimation to 8 °C and 32 °C, compared to fish at 16 °C and 24 °C [28,50]. Further, in a recent study, European seabass acclimatized to elevated temperatures (33 °C) for 10 days demonstrated significantly higher levels of *hsp70* expression compared to fish at 22 °C [28,60]. Moreover, European seabass exposed to 8 °C for 20 days prior to acclimation at various hyposalinities exhibited significant upregulation of *hsp70* compared to those maintained at 22 °C [28,60].

### 2.4. Hematological and Biochemical Parameters Adaptations

Temperature plays a significant role in influencing hematological and biochemical responses in fish [61]. Studies have shown that elevated temperature stress can affect hematological parameters, including blood cell counts and morphology [28,69]. Hematological responses to temperature stress are often reflected through alterations in cellular and nuclear morphology [28]. These changes result in lipid miscibility, increasing thermo-stability as part of the general cellular stress response mechanism [70,71,72]. For instance, striped catfish (*Pangasianodon hypophthalmus*) exposed to 36 °C for 7 days exhibited significant reductions in Hb and RBC content compared to those at 28 °C, while WBC showed the opposite trend [72]. In Indian major carp, rohu (*Labeo rohita*), Ashaf-Ud-Doulah et al. [70] reported significant changes in hematological parameters under higher temperature conditions (36 °C). Specifically, there was a marked increase in white blood cell (WBC) counts, accompanied by a significant rise in neutrophil numbers and a decrease in lymphocyte counts at the highest temperature tested (36 °C).

Additionally, temperature stress can alter blood cell counts and cellular morphology by affecting lipid layers and energy storage. Several studies have documented erythrocytic cellular and nuclear abnormalities caused by temperature fluctuations [44,70,73]. Shahjahan et al. [72] observed high-temperature effects on erythroblasts (Ebs), erythrocytic cellular abnormalities (ECA), and erythrocytic nuclear abnormalities (ENA) in striped catfish exposed to 28 °C, 32 °C, and 36 °C for 7 days, with elevated frequencies of Ebs, ECA, and ENA at higher temperatures. In a separate study, striped catfish exposed to 24 °C, 28 °C, 32 °C, and 36 °C for 28 days showed significantly higher ECA and ENA at the highest temperature [73]. Similarly, European seabass exhibited significantly higher frequencies of ECA and ENA at temperatures of 8 °C and >32 °C compared to 16 °C and 24 °C during a 10–30 day exposure period [28,43]. In red-spotted grouper (*Epinephelus akaara*), exposure to higher water temperatures (31 °C and 34 °C) for 42 days resulted in significantly decreased erythrocyte major axis and nucleus major axis compared to those at 25 °C and 28 °C [69].

During temperature stress, the release of specific cellular enzymes into the bloodstream can serve as stress indicators, reflecting damage to functional tissues and organs [74]. In catfish (*Horabagrus brachysoma*) exposed to 33 °C and 36 °C for 30 days, significant increases in LDH, malate dehydrogenase (MDH), alkaline phosphatase (ALP), acid phosphatase (ACP), AST, and ALT activities were observed, along with decreased RBC, hematocrit, Hb, mean corpuscular volume, and mean corpuscular hemoglobin [75].

### 2.5. Immune System Adaptation

Temperature extremes, both acute and chronic, significantly impair the immune functions of fish, reducing their ability to combat pathogens [28,76]. One of the primary features of immune responses to temperature stress is the elevation of antibody levels [27,28,76,77,78]. Serum lysozyme (LSZ) activities are vital innate immune defense molecules in teleosts responsible for breaking down the cell walls of both Gram-positive and Gram-negative bacteria [28,79]. Lysozyme is opsonic, activating the complement system and promoting phagocytosis [28,77,79]. Immunoglobulins (Ig) or antibodies, which play a vital role in adaptive immune responses, are heterodimeric glycoproteins belonging to the broad Ig superfamily (IgSF) with Immunoglobulin M (IgM) being the most predominant found in fish [28,80,81]. Hepcidin, a cysteine-rich antimicrobial peptide produced and secreted by the liver during inflammation, plays a key role in antimicrobial defense [28,82]. Furthermore, interleukin (IL)-1β and tumor necrosis factor-alpha (TNF-α) are significant mediators of the inflammatory response in fish [28,83,84]. For example, turbot (*Scophthalmus maximus*) exposed to high temperatures (20 °C, 23 °C, 25 °C, 27 °C, and 28 °C) resulted in a significant increase in mucus immunity, including IgM, IL-1β, hepcidin, transferrin, LSZ, and acid/alkaline phosphatase [4]. Another study reported a significant decrease in LSZ activity in Nile tilapia reared at 33 °C after 4 weeks of exposure [85]. Significantly increased plasma IgM and LSZ were also reported for olive flounder exposed at 28 and 30 °C for 2 weeks [50].

Gill and liver lysozyme activities, TNF-α, IL-1β, IL-8, IL-10, Hepcidin-1, 2, and transforming growth factor (TGF-β1) were significantly lower in largemouth bass (*Micropterus salmoides*) stressed at 26 °C compared to fish at 20 °C [86]. A study on turbot reported increased IgM at 17 °C, 20 °C, 27 °C, and 28 °C after 7 days of exposure, indicating the importance of temperature as an immune competence driver [87]. Shortnose sturgeon (*Acipenser brevirostrum*) showed upregulation of interferon regulatory factors (IRF 1 and 2) during high temperature exposure [88].

These findings highlight that extreme temperature events lead to diverse outcomes in immune responses, altering interactions between hosts and pathogens. Fish become more susceptible to potential pathogens and diseases due to reduced immunity at both extreme cold and warm temperatures. Although trends in immune responses have been observed in fish under temperature stress, there are still exceptions and contradictory reports. Extra caution should be exercised when interpreting immune responses during extreme temperature events in fish [28].

### 2.6. Adaptive Shifts in Ionic Balance

Fish in saline environments typically gain ions, while those in freshwater tend to lose electrolytes [28,87,89,90,91]. During extreme temperature events, ion transportation relies more on metabolic energy than on passive ion diffusion between the fish’s body and the surrounding environment, which can lead to osmotic failure [28,92,93,94]. Temperature changes reduce ion influx in freshwater fish, leading to net ion loss, while in seawater fish, the reverse occurs. To compensate for ion loss (in freshwater) or ion gain (in saltwater), fish increase Na^+^-K^+^ ATPase and Na^+^-K^+^-Cl^−^ cotransporter (NKCC) activity in their gills, kidneys, and intestines. Additionally, fish reduce epithelial permeability in freshwater to slow down ion loss, while it increases in saltwater to enhance ion outflow [28,89,91,94,95]. As a result, ionic imbalances can compromise central nervous system (CNS) function, impairing synaptic transmission efficiency and provoking primary stress responses, which affect all physiological functions in fish [28,91,96].

Temperature affects gill plasticity, notably impairing Na^+^-K^+^ ATPase activity. Typically, Na^+^-K^+^ ATPase activity decreases at low temperatures and increases at higher temperatures [28,91,97,98]. Na^+^-K^+^ ATPase activity is crucial for branchial and renal chloride cells, which regulate Na^+^ and Cl^−^ uptake based on electrochemical gradients between blood and the surrounding water [95,98,99]. For example, European seabass exposed to 33 °C from 24 °C showed increased Na^+^, Cl^−^, and K^+^ concentrations [28,100]. Vargas-Chacoff et al. [91] studied the interaction of temperature and salinity on osmotolerance and gill osmoregulatory proteins in Atlantic salmon, *S. salar* smolts. Fish exposed to warm stress in freshwater (FW) and seawater (SW) for 8 days at temperatures ranging from 14 to 17, 20, and 24 °C showed 100% mortality in SW at 24 °C, while no mortality occurred in other groups. Na^+^-K^+^-ATPase and NKCC activities increased in SW fish compared to FW fish but decreased at the higher temperature (24 °C) in both high and low salinities. In milkfish (*Chanos chanos*), hypotemperature stress (18 °C) for 21 days resulted in increased Na^+^, K^+^-ATPase and 11β-hydroxysteroid dehydrogenase 1 and 2 (11β-Hsd1 and 2) activity in gills compared to fish at 28 °C [28,101]. In turbot and sole (*Solea senegalensis*) acclimatized at 18 °C and 11 °C for 21 days, plasma Na^+^ and Cl^−^ concentrations were significantly reduced compared to fish at 4 and 0 °C [102]. Bernard et al. [103] found increased Na^+^-K^+^ ATPase activity and plasma Na^+^ and Cl^−^ in Atlantic salmon, reared at 5 °C and 8 °C compared to those at 15 °C and 20 °C after 90 days. European seabass exposed to 8 °C and 32 °C showed significant upregulation of Na^+^-K^+^ ATPase, NKCC1, and CFTR (Cystic fibrosis transmembrane conductance regulator) in both low and high saline waters [28,60,61,100].

### 2.7. Growth and Reproductive Adaptation

Environmental temperatures influence feed intake, digestion, absorption, and assimilation in ectotherms [28,104,105,106,107]. Fish allocate extra energy to regain homeostasis during extreme temperature events. Under non-stress conditions, energy supply is proportionally distributed for maintenance, growth, development, activities, and storage. However, during stressful situations, energy is diverted to cover additional energy requirements for stress minimization and cellular repair, reducing the energy available for growth, development, immunity, and reproduction. Consequently, growth, reproduction, and immune performance are negatively impacted. Growth inhibition and changes in reproductive performance and sex ratios during temperature stress have been documented [26,28,60,61,108]. However, some teleosts within their preferred temperature range may experience benefits from temperature increases within the aerobic scope [109,110]. Mild temperature increases (eustress) result in higher metabolic activities, improved absorption, assimilation rates, and faster physiological responses, leading to higher growth rates [111]. Conversely, laboratory-controlled temperature studies have shown that growth performance diminishes once the temperature reaches a critical threshold [29,44,77,112,113]. Reduced growth, feed intake, and food conversion efficiency are common during temperature stress. For instance, Atlantic salmon showed decreased growth performance at 17 °C and 22 °C compared to 10 °C during a 5-month study [114].

Changes in ambient water temperatures, among other factors, have been found to affect spawning, reproduction, sexual maturity, and sex differentiation by disrupting steroidogenesis and gametogenesis [28,111,115,116]. Teleost reproductive performance is expected to be increasingly affected by climate change-induced temperature fluctuations [5,116,117]. Temperature changes can either advance or delay gametogenesis and maturation via endocrine disruption [29]. Exposure of Atlantic salmon to higher temperatures during gametogenesis impaired steroid synthesis in gonads, liver vitellogenin production, and hepatic estrogen dynamics [5]. These changes can lead to reduced reproductive investment and gamete viability [118]. Elevated temperatures during gonadal maturation phases disrupt steroidogenesis, delay pre-ovulatory shifts, or inhibit them entirely [111,118,119]. Similarly, in Northern pike (*Esox lucius*), elevated temperature exposure has inhibited spermiation and negatively affected sperm motility and quality [120].

## 3. The Role of GABA in Stress Mitigation and Implications for Temperature Stress

GABA is a potent bioactive compound and an inhibitory neurotransmitter in the central nervous system [121,122], known for its efficacious role in alleviating stress responses [123]. Previous studies have shown that GABA has a potential positive effect on stress response by modulating human brain wave activity (α-wave increase and β-wave decrease) to promote relaxation and reduce anxiety, as well as enhancing immune responses to reduce stress [122,124]. In fish, GABA has emerged as a critical mediator for alleviating physiological disruptions caused by environmental stressors, including crowding, hypoxia, and handling [21,125]. These findings lay the groundwork for exploring its potential applications in mitigating temperature stress, a significant challenge in aquaculture due to climate-induced variability in water temperature.

### 3.1. GABA’s Role in Stress Mitigation

When organisms are exposed to environmental stressors, such as temperature fluctuations, the hypothalamus responds by secreting corticotropin-releasing hormone (CRH). CRH, in turn, acts on the pituitary gland to stimulate the production and release of adrenocorticotropic hormone (ACTH) into the circulation [126]. Circulating ACTH then induces the adrenal gland to synthesize and release corticosteroids, such as cortisol and corticosterone. These circulating corticosteroids modulate the vast array of physiological processes influenced by the Hypothalamus-Pituitary-Adrenal (HPA) axis and are also responsible for initiating a negative feedback loop on the HPA axis via activation of the glucocorticoid receptor (GR) in the brain to shut down corticosteroid production [127]. This response is part of the organism’s acute stress reaction, enabling it to cope with immediate challenges. However, prolonged activation of the HPA axis can lead to a hypercortisolic state, which suppresses immune function, impairs metabolic processes, and ultimately contributes to long-term health issues [128,129].

**Figure 2:**
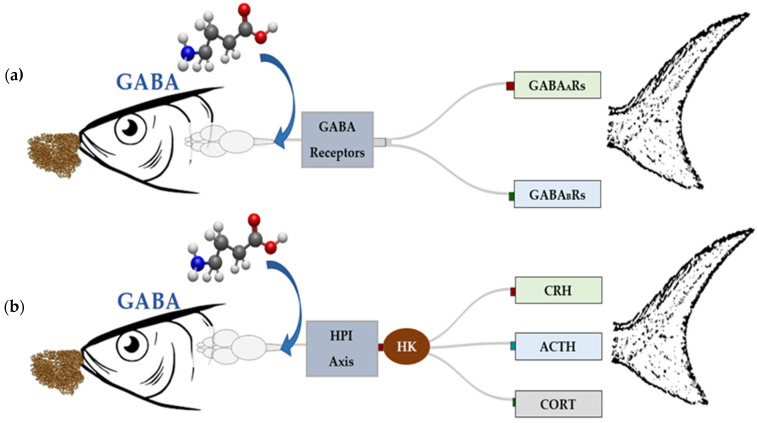
Key components of the stress response and GABAergic signaling in fish include GABA. (**a**) Upon consumption of GABA-supplemented feed, GABA interacts with two receptor types: GABA_A_Rs (GABA Type A Receptors), which are ionotropic ligand-gated chloride channels mediating fast-acting inhibitory responses, and GABA_B_Rs (GABA Type B Receptors), which are metabotropic G protein-coupled receptors producing slower and prolonged inhibitory effects. (**b**) Following feed intake, dietary GABA also acts through the HPI axis, analogous to the HPA axis in higher vertebrates, governs the endocrine stress response in fish, and connects the hypothalamus, the pituitary gland, and the head kidney (where interrenal cells are located). In this system, GABA influences the HPI axis, where it inhibits the release of CRH, ACTH, and cortisol. The arrows indicate the direction of GABA action and flow of regulatory signaling leading to stress modulation.

In fish, the hypothalamus-pituitary-interrenal (HPI) axis functions similarly as the hypothalamus-pituitary-adrenal (HPA) axis found in higher vertebrates, with the only difference being the replacement of the adrenal glands with the interrenal cells located in the head kidney of fish. By modulating the hypothalamic-pituitary-interrenal (HPI) axis [130] (Figure 2), GABAergic neurons help control cortisol levels and mitigate stress-induced damage. This regulation is achieved by inhibiting the release of corticotropin-releasing hormone (CRH) and adrenocorticotropic hormone (ACTH), two essential hormones for cortisol activation [131]. This inhibitory mechanism protects organisms from the detrimental effects of chronic or temperature-induced stress, promoting overall physiological well-being [132]. Disruptions in the regulation of the HPI axes can result in abnormal stress responses [133]. The adaptability of the GABAergic system allows the brain to dynamically respond to environmental stressors, maintaining neural stability and help organisms cope with both acute and chronic stress. In addition to modulating the hypothalamic-pituitary-interrenal (HPI) axis, GABA plays a critical role in regulating neuronal excitability, which is essential for the brain’s overall response to stress [134,135].

This function is mediated through activation of two classes of receptors with distinct electrophysiological and pharmacological properties. Type A GABA receptors (GABA_A_Rs) are ionotropic fast-acting ligand-gated chloride channels [136,137], while type B GABA receptors (GABA_B_Rs) belong to the metabotropic G protein-coupled receptor superfamily and produce slow and prolonged inhibitory responses [138,139]. GABA_A_Rs are heteropentamers composed of various combinations of 19 subunits, including six α (alpha1-6), three β (beta1-3), three γ (gamma1-3), three ρ (rho1-3), and one each of the δ (delta), ε (epsilon), π (pi), and θ (theta) subunits, which collectively produce a wide array of receptor isoforms (140, 141). These isoforms exhibit distinct pharmacological and physiological properties, with the majority of GABA_A_Rs comprising two α subunits, two β subunits, and one γ subunit, arranged as γ2β2α1β2α1 counterclockwise around the receptor’s center [140,141]. However, GABA_A_Rs are the primary mediators of fast inhibitory neurotransmission in the central nervous system, which is pivotal in terminating stress-induced neural hyperactivity [141]. By stabilizing neuronal excitability and preventing overactivation of stress-related pathways, GABA_A_Rs mitigate the adverse effects of both acute and chronic stress, ensuring a balanced and adaptive response to environmental challenges. Dysregulation of GABA_A_Rs signaling can impair this critical process, leading to maladaptive stress responses and associated health consequences [140,142].

During stress, the activation of the HPI axis and the sympathetic–adrenal–medullary (SAM) system induces rapid changes in GABA_A_Rs function [143]. These changes are mediated through processes such as the release of neurosteroids, which act as positive modulators of GABA_A_Rs, enhancing their inhibitory effects [144]. Additionally, GABA_A_Rs trafficking, the dynamic relocation of receptors within the neuronal membrane, enables the brain to adjust inhibitory signaling in response to stress swiftly [141,142,145]. This receptor trafficking allows the brain to adapt quickly to changing environmental conditions, such as temperature fluctuations, by regulating GABAergic signaling to maintain neural stability [146]. These rapid adjustments in GABA_A_Rs function are particularly vital during temperature-induced stress, where prompt physiological responses are required to maintain homeostasis.

These combined mechanisms of GABAergic regulation support the brain’s ability to adapt to stress, enabling organisms to cope with environmental changes, including temperature fluctuations. GABA’s dual role in modulating both the neuroendocrine response and neuronal excitability underscores its effectiveness in mitigating temperature-induced stress. By reducing cortisol secretion, stabilizing neural activity, and enhancing the organism’s ability to respond to environmental changes, GABA emerges as a powerful tool for managing the physiological impacts of temperature stress.

This has significant implications for aquaculture, where rising water temperatures are an increasing concern. GABA supplementation presents a promising strategy to help fish manage the effects of temperature stress, safeguarding their health, growth, and productivity in the face of climate-induced environmental challenges. As aquaculture practices adapt to global climate change, the potential of GABA to mitigate the detrimental effects of temperature stress offers exciting possibilities for enhancing the resilience of aquaculture species. While GABA’s role in improving fish resilience to fluctuating water temperatures is promising, limited studies have directly addressed its effects on temperature-induced stress. Therefore, examining its well-documented role in alleviating non-temperature-related stressors may provide valuable insights into its broader applications, including temperature stress mitigation in aquaculture.

### 3.2. GABA-Mediated Signaling Pathways in Temperature Stress Regulation

**Figure 3:**
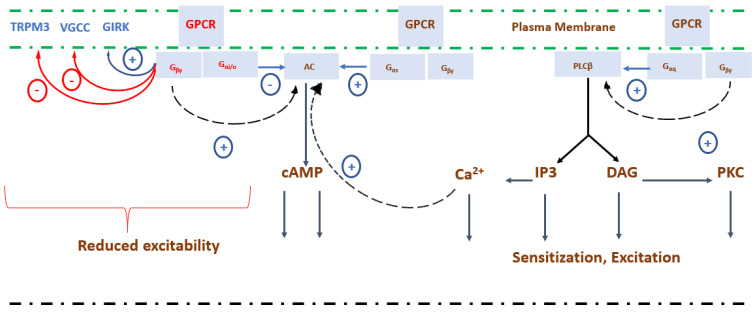
Proposed intracellular signaling pathways mediated by G protein-coupled receptors (GPCRs) and their effects on cellular excitability. Activation of GPCRs at the plasma membrane triggers distinct intracellular cascades through various G protein subunits. The Gαi/o and Gβγ subunits inhibit transient receptor potential (TRPM) channels, voltage-gated calcium channels (VGCCs), and activate G protein-regulated inwardly rectifying potassium (GIRK) channels, leading to reduced neuronal excitability (red pathways). Conversely, Gαs subunits stimulate adenylyl cyclase (AC), increasing cyclic adenosine monophosphate (cAMP) levels and enhancing calcium (Ca^2+)^ influx, thereby promoting excitatory responses. The G_αq_ subunits activate phospholipase C-β (PLCβ), generating inositol trisphosphate (IP3) and diacylglycerol (DAG), which subsequently increase intracellular Ca^2+^ and activate protein kinase C (PKC), leading to cellular sensitization and excitation (black pathways). Together, these mechanisms illustrate the opposing roles of GPCR-mediated signaling in modulating excitability and stress-related neurotransmission in fish neurons. Arrows indicate the direction of signal flow, while positive (+) and negative (-) symbols represent stimulatory and inhibitory effects, respectively. Adapted from Yudin and Rohacs, 2018 [168].

Beyond the neuroendocrine role of GABA described in Section 3.1, GABA-mediated thermotolerance is orchestrated through a dynamic interplay of molecular, cellular, and systemic mechanisms that stabilize neuronal excitability, reinforce cytoprotective pathways, and adjust neuroendocrine outputs during stress [147]. At the molecular level, the GABA_B_ receptor is a G-protein-coupled receptor (GPCR) that associates specifically with pertussis toxin-sensitive Gi/o family proteins, which in turn regulate ion channels and intracellular cyclic adenosine monophosphate (cAMP) cascades (Figure 3). This heterotrimeric complex consists of an α-subunit (Gαi/o) and a tightly associated βγ dimer (Gβγ). In the resting state, Gα is bound to guanosine diphosphate (GDP) and remains associated with the receptor and the Gβγ dimer. Upon receptor activation, the GABA_B_ receptor functions as a guanine nucleotide exchange factor, catalyzing the replacement of GDP with guanosine triphosphate (GTP) on Gα, which induces a conformational shift that causes the Gα–GTP and the Gβγ dimer to dissociate. Both units then independently regulate downstream effectors, including cAMP-dependent signaling pathways and ion channel activity [148,149].

The Gα–GTP subunit primarily inhibits adenylyl cyclase, reducing intracellular cAMP levels and decreasing protein kinase A (PKA) activity. This reduction relieves inhibitory constraints on the phosphatidylinositol 3-kinase (PI3K)/AKT (protein kinase B, a central serine/threonine kinase) pathway, facilitating AKT activation [150]. Activated AKT phosphorylates glycogen synthase kinase 3 beta (GSK3β), thereby relieving its inhibitory effect on heat shock factor 1 (HSF1). Once relieved, HSF1 translocates to the nucleus, binds heat shock elements, and drives the transcription of molecular chaperones, including *hsp70*, *hsp90*, and small hsps. These chaperones collectively preserve proteostasis during hyperthermia [151]. Supporting the importance of post-translational regulation, Jin et al. [152] demonstrated in mammalian cells that loss of phosphorylation at HSF1 residues S303 and S307 (serines at positions 303 and 307) increases protein stability while sensitizing HSF1 activation under both basal and stress conditions. This finding underscores how phosphorylation status can fine-tune HSF1 responsiveness, suggesting that similar regulatory mechanisms may operate in teleosts to optimize heat shock protein induction during thermal stress.

In parallel, AKT activation and endoplasmic reticulum (ER) stress promote nuclear accumulation of Nrf2 (nuclear factor erythroid 2-related factor 2), a transcription factor that binds antioxidant response elements (AREs) to drive the expression of cytoprotective genes. Nrf2 activation upregulates antioxidant enzymes such as superoxide dismutase (SOD1/2), catalase (CAT), and glutathione peroxidase, thereby enhancing cellular resilience to oxidative stress [153,154]. The role of Nrf2 in activating antioxidative defense is conserved across taxa: in molluscs such as *Crassostrea gigas*, *Mytilus coruscus*, *Ruditapes philippinarum*, and *Cristaria plicata*, high-temperature stress stabilizes Nrf2, promotes its nuclear translocation, and induces transcription of SOD and CAT [155,156,157,158]. In vertebrates, Nrf2 regulation involves proteasomal degradation mediated by Kelch-like ECH-associated protein 1 (Keap1), which maintains low basal Nrf2 levels under normal conditions and allows rapid increases in response to stress. Experimental evidence in juvenile olive flounder (*Paralichthys olivaceus*) and Pacific oyster (*Crassostrea gigas*) demonstrates that dietary GABA enhances both *hsp* and antioxidant enzyme expression under elevated temperatures, providing a mechanistic basis for GABA-mediated thermoprotection [151,158].

Simultaneously, the liberated Gβγ dimer modulates neuronal excitability and postsynaptic inhibition through direct regulation of G-protein–gated inwardly rectifying potassium (GIRK) channels and voltage-gated calcium channels (VGCCs) [159,160]. Activation of GIRK channels hyperpolarizes neurons, whereas inhibition of VGCCs reduces calcium influx, together preventing excitotoxicity during thermal stress and complementing the Gαi/o-mediated suppression of cAMP and PKA. GIRK channels are also expressed on presynaptic membranes, but their contribution to presynaptic function remains incompletely understood [161]. In some cases, GIRK activation can inhibit the release of glutamate and GABA in the CNS [160,162], yet the mechanisms by which GIRK channels regulate neurotransmitter release, modulate acetylcholine (ACh) secretion from peripheral nerves, or influence the timing of neurotransmitter release, critical for synaptic plasticity and efficient neurotransmission, remain unclear [160,163]. These presynaptic roles complement the well-characterized postsynaptic actions. The ionotropic and metabotropic mechanisms converge with GABA_A_ receptor-mediated chloride influx, generating both phasic and tonic inhibition depending on synaptic and extrasynaptic receptor localization [164]. The dynamics of GABA transporters, including GAT1–3 (which account for the largest proportion of GABA uptake in the CNS) and the betaine GABA transporter (BGT1), further regulate extracellular and intracellular GABA concentrations, modulating inhibitory efficacy [165].

At the circuit and systemic levels, these molecular and cellular processes converge on the hypothalamic–pituitary–interrenal (HPI) axis, where GABAergic inhibition limits corticotropin-releasing hormone (CRH) and adrenocorticotropic hormone (ACTH) secretion, thereby attenuating cortisol production by interrenal cells. By moderating cortisol release, GABA preserves energy stores, protects immune function, and minimizes the physiological costs of prolonged stress [166]. Experimental evidence in olive flounder demonstrates that dietary GABA supplementation suppresses cortisol spikes during acute thermal stress, enhances growth performance, and improves feed conversion [151]. Parallel studies in *Takifugu flavidus* confirm that GABA improved plasma biochemistry, energy metabolism, and antioxidant capacity [167].

Integration of these mechanisms reveals a multi-level network in which Gαi/o–PI3K/AKT–HSF1/Nrf2 signaling, chloride-dependent inhibition, and systemic neuroendocrine regulation converge to maintain homeostasis under environmental stress. GABAergic neurons are uniquely able to alter the excitability of local circuits within a given brain region. The cellular mechanisms that regulate the cell surface accumulation are under active investigation [137]. Feedback loops driven by reactive oxygen species, intracellular chloride shifts, or cortisol elevations dynamically adjust receptor expression, transporter activity, and downstream signaling, enabling adaptation to fluctuating temperatures. Dietary GABA exploits this intrinsic plasticity to enhance thermotolerance at molecular, cellular, and systemic scales in teleosts. Yet key questions persist, including the balance of phasic versus tonic GABA_A_ inhibition, receptor-subtype specificity, and the interplay between GABAergic and other neuromodulatory systems under heat stress. Addressing these gaps with receptor-selective pharmacology, genome editing, in vivo ion imaging, and integrated omics will be essential for linking molecular pathways to organismal thermotolerance, with direct implications for aquaculture resilience under climate change.

### 3.3. GABA’s Role in Non-Temperature Stress Conditions and Its Potentials for Temperature Stress Mitigation in Aquaculture

Studies have shown that GABA supplementation can significantly reduce oxidative damage, improve metabolic functions, and enhance immune responses under various stress conditions, including ammonia toxicity, transportation stress, and nutritional deprivation (Table 2). The mechanisms through which GABA exerts these benefits, such as antioxidant modulation, immune enhancement, and stress response regulation, share similarities with those needed to manage temperature stress. By drawing parallels between GABA’s effects in non-temperature stress conditions, we can gain insights into its possible role in enhancing stress resilience and managing temperature-induced challenges in aquaculture species, particularly under increasing threats posed by the rising global water temperatures.

Many aquaculture species have been reported to respond positively to GABA supplementation when subjected to various stress conditions. For instance, in largemouth bass, Sun et al. [86] showed that GABA supplementation, whether administered through diet or immersion, effectively reduced oxidative damage under ammonia stress. This highlights GABA’s capacity to bolster oxidative defense mechanisms, a benefit that may also be relevant under temperature stress, which exacerbates ammonia toxicity through increased metabolic activity and reduced detoxification efficiency.

The benefits of GABA extend beyond ammonia-related stressors. In *Takifugu flavidus*, Yu et al. [167] demonstrated that GABA immersion alleviated the physiological impacts of transportation stress by enhancing energy metabolism, boosting antioxidant capacity, and reducing stress-induced damage. This is particularly relevant to thermal stress, as transportation often involves sudden temperature fluctuations that exacerbate handling stress. By improving energy metabolism and minimizing oxidative damage, GABA may play a vital role in protecting fish health during temperature-related challenges associated with transport. However, not all species exhibit the same level of responsiveness to the same type and intensity of stress. In crucian carp, Yan et al. [122] reported that dietary GABA supplementation provided limited protection under chronic ammonia stress, as indicated by elevated oxidative stress markers and decreased antioxidant gene expression. These findings underscore the importance of a species-specific approach when applying GABA in aquaculture, particularly under complex stress conditions such as prolonged thermal or ammonia exposure.

GABA’s potential also extends to nutritional stress scenarios, such as fasting and dietary imbalances. Zhang et al. [148] reported that GABA supplementation improved glucose homeostasis and hepatopancreas function in *Eriocheir sinensis* under fasting stress, indicating its capacity to support fish during temperature-induced feeding challenges. Elevated temperatures are known to impair feeding behavior and metabolic efficiency in aquaculture species [106], suggesting that GABA could help stabilize glucose levels and enhance metabolic resilience in such conditions. These findings highlight GABA’s ability to support intestinal health and nutrient absorption, which could be particularly beneficial during thermal stress when digestion and nutrient uptake are often compromised.

Pathogen-induced stress is another area where GABA has shown promise. In *Litopenaeus vannamei*, Bae et al. [12] reported that GABA, when combined with antibiotics, improved survival rates during *Vibrio alginolyticus* infection. Similarly, Farris et al. [13] found that GABA supplementation in Paralichthys olivaceus enhanced immune responses against *Edwardsiella tarda* and *Streptococcus iniae*, highlighting its immunomodulatory potential. As temperature stress is known to compromise immune function, these findings suggest that GABA could mitigate thermal stress by bolstering immune resilience. In oxygen-limited environments, such as those caused by temperature-induced hypoxia, GABA has also demonstrated utility. Varghese et al. [125] observed that GABA improved metabolic functions in *Cirrhinus mrigala* under hypoxic conditions. Warmer water holds less dissolved oxygen, and the ability of GABA to enhance energy metabolism under these conditions may make it a valuable tool for aquaculture species facing combined thermal and hypoxic stress. In the case of density stress, Bae et al. [22] explored GABA supplementation in olive flounder, demonstrating its efficacy in enhancing immune response under combined density stress and pathogenic challenges. Although GABA did not produce a significant response under acute density stress in olive flounder, its benefits were evident under chronic density stress. This highlights the need to identify the factors that may influence GABA’s efficacy across different stress scenarios.

## 4. Factors Influencing GABA-Mediated Stress Response

**Figure 4:**
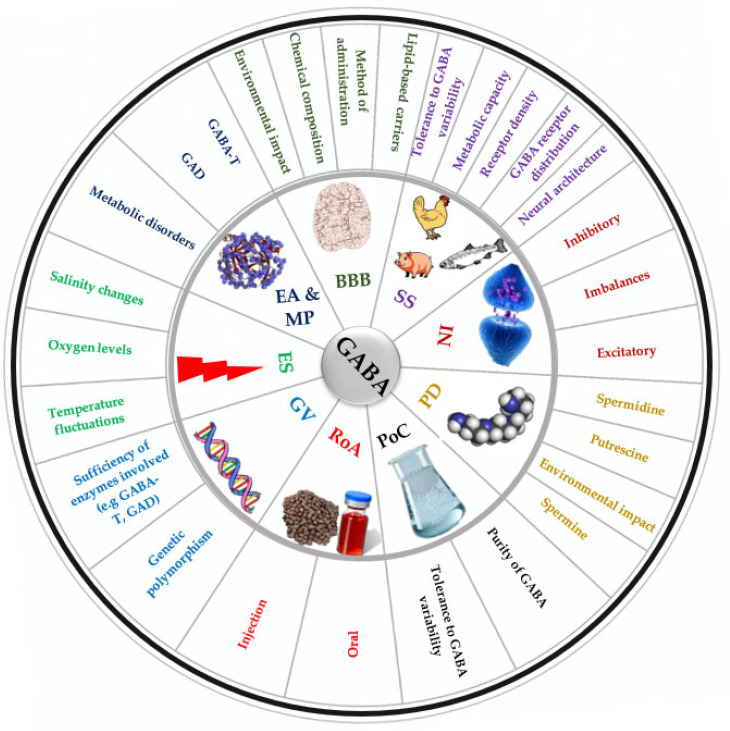
The factors influencing the efficacy of GABA in fish. It is divided into concentric layers, with the innermost layer representing key categories such as Blood–Brain Barrier (BBB) permeability, route of administration, and species-specific variations. The outermost segments represent specific factors that match the innermost layer that contribute to GABA’s physiological and biochemical actions. The chart highlights the complex interactions between genetic, environmental, and biochemical factors that modulate GABA’s effectiveness in modulating stress and other physiological responses in fish, emphasizing the role of various internal and external variables in determining its efficacy. NI: Neurotransmitter Interaction; EA & MP: Enzyme Activity and Metabolic Pathway; GV: Genetic Variation; SS: Species Specificity; RoA: Route of Administration; PD: Polyamine Degradation; PoC: Purity of Chemical; ES: Environmental Stress; BBB: Blood-Brain-Barrier; GABA-T: Gaba transport; GAD: Glutamate decarboxylase.

The GABA-mediated stress responses in fish are affected by several factors [50] (Figure 4). Understanding these factors is crucial for researchers developing effective GABA-related dietary interventions for various fish species.

For instance, GABA has been shown to have the ability to cross the blood–brain barrier (BBB), potentially through transporter systems in the brain such as the passage of solutes via transcytosis, carrier-mediated transport, or simple diffusion of hydrophobic substances [10,51,170,171], contrary to earlier belief that the BBB is impermeable. These divergent opinions stem from variations in chemical compounds and methods of administration (i.e., oral versus injection) employed in their research. The route of administration of GABA can significantly determine its effectiveness and physiological impact [170]. For instance, whether GABA is administered orally or through injection can influence its bioavailability and ability to cross the BBB, potentially affecting its efficacy in modulating neurotransmission and stress responses [170]. To address the challenge of GABA’s limited bioavailability, researchers in other fields have explored several advanced technologies. Lipid-based carriers, such as liposomes, can enhance GABA’s bioavailability by facilitating its transport across biological membranes. Biodegradable polymer-based systems, like chitosan nanoparticles, may offer controlled release of GABA over extended periods, improving its efficacy. Additionally, strategies like using prodrugs or chemical modifications to increase GABA’s lipophilicity may show promise in enhancing its ability to cross the blood–brain barrier (BBB) just like Lipid-core nanocapsules (LNCs), for instance, have been demonstrated to effectively transport drugs across the BBB, increasing drug concentration in the brain [45].

Furthermore, species specificity plays a critical role in determining GABA’s effect on stress responses. Fish with different neural architectures or variations in GABA receptor density and distribution may exhibit distinct stress responses [35]. For example, species with a higher density of GABA receptors in specific brain regions might experience more pronounced inhibitory effects from GABA, leading to a greater reduction in stress-related behaviors and physiological markers [172]. GABA responses could be mediated by either GABA receptors or GABA transporters [56]. In Prussian carp (*Carassius gibelio*), Snigirov and Sylantyev [173] discovered that feeding activity is regulated by GABA receptors through two distinct mechanisms: orthosteric and allosteric. The orthosteric mechanism involves the direct binding of GABA to its receptor, which triggers a response. In contrast, the allosteric mechanism does not depend on GABA; instead, it involves the binding of other compounds, such as neurosteroids, to sites on the GABA receptor that are separate from the GABA binding site [44]. As a result, even slight changes in neurosteroid levels can significantly affect the GABAergic regulation of neuronal excitability [174]. Moreover, GABA_A_ receptors (GABA_A_Rs) are particularly sensitive to neurosteroid modulation at higher concentrations [44,60,61]. This interaction enhances the receptors’ response to GABA, leading to a more pronounced influence on feeding behavior and overall physiological responses in fish.

As part of species specificity as a crucial factor influencing GABA’s efficacy, genetic variations among different species influence the enzymatic processes involved in the synthesis of GABA (such as glutamate decarboxylase) and the metabolism of GABA (such as GABA transaminase), ultimately affecting the overall levels of GABA in the brain and its availability for neurotransmission [174]. The GABA shunt relies on many enzymatic pathways [60,61]. Deficiencies in enzymatic activity resulting from genetic mutations or changes in expression levels (either upregulation or downregulation) can lead to various metabolic disorders that may simultaneously impact multiple metabolic pathways [42]. Inherited metabolic disorders can arise from mutations in genes involved in the biogenesis, assembly, or activity of metabolic enzymes, leading to enzymatic deficiencies and severe, life-threatening metabolic impairments [62,175]. This variability can contribute to individual differences in GABAergic neurotransmission and may affect neurological functions. The upregulation or downregulation of metabolic enzymes can promote and sustain the activation of metabolic pathways [44].

**Figure 5:**
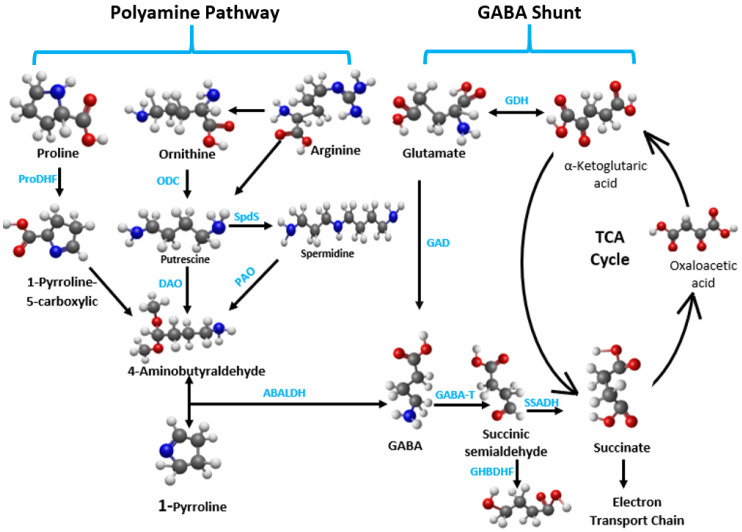
Three-dimensional representation of the biosynthetic and metabolic pathways of γ-aminobutyric acid (GABA) and related polyamines. Adapted from Rashmi et al. [176]. The figure was realized using an open-source molecular builder and visualization tool, Avogadro version 2.0 previously used by Ncho et al. [177]. Carbon atoms are represented in black; hydrogen atoms in white; nitrogen atom in blue; oxygen atoms in red. The enzymatic conversions and metabolic interconnections between amino acids, polyamines, and the tricarboxylic acid (TCA) cycle. The figure integrates both the synthesis and degradation pathways of GABA, highlighting the role of polyamines such as putrescine, spermidine, and spermine in the generation of GABA through oxidative deamination reactions. ProDH, proline dehydrogenase; ODC, ornithine decarboxylase; SpdS, spermidine synthase; DAO, diamine oxidase; PAO, polyamine oxidase; ABALDH, 4-aminobutyraldehyde dehydrogenase; GAD, glutamate decarboxylase; GDH, glutamate dehydrogenase; GABA-T, GABA transaminase; SSADH, succinate semialdehyde dehydrogenase; GHBDHF, γ-hydroxybutyrate dehydrogenase form.

The interaction between GABA and other neurotransmitters, such as glutamate, adds another layer of complexity. Glutamate, a major excitatory neurotransmitter in the brain, serves as a precursor for GABA and is converted to GABA through a decarboxylation reaction (Figure 5). Thus, there is a direct relationship between glutamate content and GABA content [106]. The balance between excitatory (glutamate) and inhibitory (GABA) signals is essential for maintaining neural homeostasis. Any disruption in this balance, potentially caused by environmental stressors or dietary factors, can influence the effectiveness of GABAergic modulation. For instance, an excess of glutamate could diminish the inhibitory effects of GABA, potentially leading to heightened stress responses. Conversely, adequate levels of both neurotransmitters can facilitate a balanced neurotransmission capacity, improving the innate stress response [58].

Another factor that affects the GABA synthesis pathway and, ultimately, its neurotransmission capabilities is polyamine degradation [106] (Figure 5). Polyamines (PAs) are aliphatic amines with low molecular mass that are ubiquitous in all living organisms, playing roles in diverse biological processes [59,174]. The main PAs include putrescine, spermine, and spermidine, with putrescine serving as the central substance in PA metabolism. Putrescine is classified as a primary PA, while spermidine and spermine are considered secondary and tertiary PAs, respectively [106]. Putrescine can be produced from ornithine, catalyzed by ornithine decarboxylase (ODC) or from arginine, catalyzed by arginine decarboxylase (ADC). Diamine oxidase (DAO) and polyamine oxidase (PAO) are the enzymes involved in PA degradation [62]. This degradation pathway refers to the process by which diamines or PAs are catalyzed by DAO and FAD-dependent PAO [62,170], respectively, to produce 4-aminobutyraldehyde (ABAL), which is subsequently dehydrogenated by 4-aminobutyraldehyde dehydrogenase (AMADH) to generate GABA [25,178]. Like glutamate, PAs also serve as precursors of GABA [25,64,65]. Adverse environmental conditions can reduce the efficiency of PA degradation, leading to an accumulation of PA [106], which can result in cell apoptosis [64]. In such situations, supporting GABA homeostasis through dietary supplementation may help mitigate the damaging effects of PA buildup. Furthermore, maintaining intracellular PA homeostasis may ultimately contribute to the preservation of oxidative homeostasis [152]. For instance, maintaining GABA homeostasis in the presence of elevated PA levels has been reported to increase osmotic plasticity and prevent gill apoptosis in killifish [64].

Furthermore, the purity levels of chemical substances can significantly impact their bioavailability across different species. Bioavailability, which refers to the potential for uptake by living organisms, is influenced by the purity of the substance and can affect its circulation and metabolism within organisms [50]. Different species may exhibit varying tolerance levels to impurities; some may utilize lower-purity compounds, while others may struggle due to their liver’s inability to break down highly pure substances [37]. Thus, understanding the purity levels of chemical substances and the degradation capacity of different fish species is essential for improving their bioavailability and assessing their potential effects. For instance, Ruenkoed et al. [15] reported that 50% pure GABA in Nile tilapia improved weight, digestive enzymes, antioxidant activity, intestinal morphology, and gene expression of immune and growth-related genes. Similar results were observed in grass carp, where 50% pure GABA enhanced both antioxidant and growth capabilities [170]. In contrast, it was ≥99% pure GABA that was used to achieve similar results in Jian carp [51], while 76.5% pure GABA did not yield significant differences in growth parameters in olive flounder [50].

These are some of the key underlying elements and complexities that influence the results reported in numerous investigations focused on mitigating stress in aquaculture species. Additional research is needed to elucidate these factors. For instance, assessing the levels of PA metabolites alongside other physiological parameters following GABA supplementation in studies aimed at evaluating stress response may provide valuable insights into the complexities of GABA’s potential for stress alleviation in aquaculture species.

## 5. Conclusions

The potential of GABA in mitigating temperature-induced stress presents exciting possibilities for aquaculture, but significant research gaps remain. While GABA is well-recognized for its ability to alleviate stress by modulating the HPI axis and regulating neuronal excitability, its specific mechanisms under temperature stress are not yet fully understood. We identified the blood–brain barrier, route of administration, purity, species specificity and polyamine degradation as key factors influencing the efficacy of the GABAergic pathway in stress mitigation

Future studies should focus on elucidating the precise biochemical pathways and receptor dynamics involved, particularly in the context of fluctuating water temperatures caused by climate change. Adoption of complementary strategies such as incorporating molecular and genetic tools could offer additional insights into GABA’s role. Transcriptomic and genomic studies could reveal key regulatory genes and pathways, paving the way for selective breeding programs aimed at enhancing thermal resilience in aquaculture species.

Finally, integrating GABA-based strategies into broader aquaculture frameworks can amplify their impact. Aligning these interventions with ecosystem-based management and climate-resilient farming practices will ensure sustainable and adaptive aquaculture systems. Addressing these research gaps will help unlock the full potential of GABA, offering a vital tool to mitigate the challenges posed by climate change and ensure the sustainability of aquaculture industries.

## Figures and Tables

**Table 2:** Summary of results from GABA-related stress experiments in aquatic organisms.

Species	Type of Stress	Route of Administration	Effect	Dosage	Duration of Stress	References
*Litopenaeus vannamei*	*Vibrio alginolyticus* challenge	Diet	Survival rate 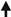	50, 100, 300 mg/kg + 4 g oxytetracyclin	9 days	[12]
*Eriocheir sinensis*	Fasting stress	Injection	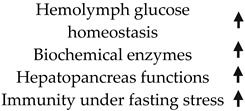	100, 1000 μmol/mL	48 h	[169]
*Paralicthyes olivaceus*	*Streptococcus iniae* challenge	Diet	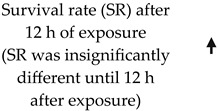	CON, with 92 mg/kg GABA content), a positive control with 4 g/kg oxytetracycline (OTC), and five other diets supplemented with 50, 100, 150, 200 and 250 mg/kg	12 days	[13]
*Litopenaeus vannamei*	Ammonia stress	Diet	SR beginning from day 11 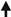 Antioxidants 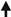	0, 50, 100, 150, 200, 250 mg/kg	36 h	[14]
*Cirrhinus mrigala*	Hypoxia	Diet	Metabolism during hypoxia 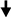	0.00%, 0.50%, 0.75%, 1.0%	72 h	[125]
*Eriocheir sinensis*	Lipopolysaccharide	Diet	Anti-lipopolysaccharide factors and anti-inflammatory signaling pathways 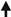	0, 40, 80, 160, 320, 640 mg/kg	24 h	[21]
*Oreochromis niloticus*	Ammonia	Diet	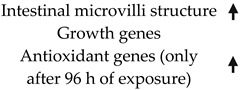	0, 200, 500 mg/kg	96 h	[15]
*Takifugu flavidus*	Transportation stress	Immersion	Plasma Biochemistry 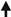 Energy metabolism 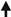 Antioxidant capacity 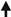	0, 5, 50, and 150 mg/L	3 days	[167]
*Paralicthyes olivaceus*	Density and *Edwardsiella tardar* challenge	Diet	Immune response 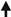	0, 150, 200, 250 mg/kg	48 h	[22]
*Carassius carassius*	Ammonia stress	Diet	Growth impairment 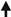 Malondialdehyde 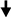 Liver injury 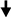 Antioxidant genes 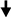	100 mg/kg	56 days	[122]
*Micropterus salmoides*	Ammonia	Immersion and Diet	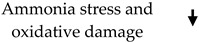	Immersion: 0, 30, 60, 90, 120, and 150 mg/L Diet: 0, 30, 90, and 150 mg/kg	Immersion: 96 h; samples taken every 24 h Diet: 15 days; samples taken every 3 days	[86]
*Eriocheir sinensis*	Hypoxia	Diet	Oxygen consumption 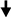 Neural excitotoxicity 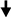 Antioxidants 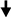	0, 250, 500 mg/kg	24 h	[169]

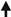
 Represent an increase in the corresponding parameter; 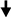 Represent an increase in the corresponding parameters.

## Data Availability

The data that support the findings of this study are available on request from the corresponding author. The data is not publicly available due to privacy or ethical restrictions.

## Abbreviations

The following abbreviations are used in this manuscript:

AChE	Acetylcholinesterase
ACP	Acid phosphatase
ACTH	Adrenocorticotropic hormone
ADC	Arginine decarboxylase
AKP	Alkaline phosphatase
ALP	Alkaline phosphatase
ALT	Alanine transaminase
AREs	Antioxidant response elements
AST	Aspartate transaminase
BBB	Blood–Brain Barrier
cAMP	cyclic adenosine monophosphate
CAT	Catalase
CHO	Cholesterol
CNS	Central nervous system
CORT	Cortisol
CRH	Corticotropin-releasing hormone
DA	Dopamine
DAO	Diamine oxidase
E2	Estradiol-17β
EA	Enzyme Activity
Ebs	Erythroblasts
ECA	Erythrocytic cellular abnormalities
ENA	Erythrocytic nuclear abnormalities
ER	Endoplasmic reticulum
ES	Environmental Stress
GABA	Gamma-aminobutyric acid
GAD	Glutamate decarboxylase
GCL	Glutamate cysteine ligase
GDP	Guanosine diphosphate
GH1	Growth hormone 1
GIRK	G-protein–gated inwardly rectifying potassium
GLU	Glucose
GPx	Glutathione peroxidase
GR	Glucocorticoid receptor
GSH	Glutathione
GST	Glutathione-S-transferase
GV	Genetic Variation
GTP	Guanosine triphosphate
HDL	High density lipoprotein
HSF1	Heat shock factor 1
HPA	Hypothalamus–pituitary–adrenal
HPI	Hypothalamus–pituitary–interrenal
*hsp*	Heat shock protein
IRF	Interferon regulatory factors
LDH	Lactate dehydrogenase
LDL	Low density lipoprotein
LNCs	Lipid-core nanocapsules
LPO	Lipid peroxidation
LSZ	Serum lysozyme
LZM	Lysozyme
MDA	Malondialdehyde
MDH	Malate dehydrogenase
MP	Metabolic Pathway
MRB	Macrophage respiratory burst
MTLP	Metallothionein-like proteins
NBT	Nitroblue tetrazolium
NE	Noradrenaline
NI	Neurotransmitter Interaction
Nrf2	Nuclear factor erythroid 2–related factor 2
ODC	Ornithine decarboxylase
PAO	Polyamine oxidase
PD	Polyamine degradation
PI3K	Phosphatidylinositol 3-kinase
PKA	Protein kinase A
PoC	Purity of Chemical
RoA	Route of Administration
ROS	Reactive oxygen species
RS & M	Receptor Sensitivity and Mechanisms
SAM	Sympathetic–adrenal–medullary
SOD	Superoxide dismutase
SS	Species Specificity
TBARS	Thiobarbituric acid
TG	Triglycerides
TGF-β1	Transforming growth factor
TNF-α	Tumor necrosis factor-alpha
VGCCs	Voltage-gated calcium channels
WBC	White blood cell
